# The importance of activated TMPRSS2 in the proviral role of small extracellular vesicles in SARS‐CoV‐2 infection

**DOI:** 10.1002/jev2.12296

**Published:** 2022-12-21

**Authors:** Sze Keong Tey, Judy Wai Ping Yam

**Affiliations:** ^1^ Department of Surgery School of Clinical Medicine Li Ka Shing Faculty of Medicine The University of Hong Kong Hong Kong China; ^2^ Department of Pathology School of Clinical Medicine Li Ka Shing Faculty of Medicine The University of Hong Kong Hong Kong China

1

**To the Editor**:

We read with great interest the article by Berry et al., showing the proviral role of human respiratory epithelial cell‐derived small extracellular vesicles (sEVs) in severe acute respiratory syndrome coronavirus 2 (SARS‐CoV‐2) infection (Berry et al., 2022). Berry et al. found that the mucus sEVs (mu‐sEVs) produced by human nasal epithelial cells (HNECs) contain SARS‐CoV‐2 receptor angiotensin‐converting enzyme 2 (ACE2) and activated protease transmembrane protease serine 2 (TMPRSS2) that cleave prefusion viral Spike (S) proteins at the S1/S2 boundary, resulting in higher proportions of prefusion S proteins with ‘open’ receptor‐binding domain that facilitates receptor binding at the cell surface to enhance virus infection.

The definite role of extracellular vesicles (EVs) in SARS‐CoV‐2 infection is still debatable. At the very beginning, the presence of ACE2 in sEVs was proposed as a novel competitive inhibition therapy against SARS‐CoV‐2 by occupying S proteins S1 domain to protect cells from infection (Inal, 2020). Indeed, emerging studies have provided evidence using authentic or engineered ACE2‐expressing sEVs to block pseudo‐ or live viruses’ infection (Ching et al., 2022; Cocozza et al., 2020; El‐Shennawy et al., 2022; Kim et al., 2022; Rao et al., 2020; Wu et al., 2022; Xie et al., 2021; Zhang, Huang, et al., 2021; Zhang, Jeppesen, et al., 2021). Contrarily, our previous study that identified ACE2‐enriched sEV represents a ‘trojan horse’ for SARS‐CoV‐2 to enter host cells, at the time of publication, remained the only study to show the opposite effect of sEV‐ACE2 in authentic SARS‐CoV‐2 infection (Tey et al., 2022). Such controversial observation might be due to several factors, including the lack of clarity in experimental details that allows fairer comparison, the use of different cell lines for infection and infection method, which have been discussed in detail in our previous study (Tey et al., 2022).

The article by Berry et al. has provided important insights into the role of sEV‐ACE2 in SARS‐CoV‐2 infection and the putative molecular mechanism. In the study, ACE2 and activated TMPRSS2 were present in the HNECs‐derived mu‐sEVs, in which the activated TMPRSS2 cleaved the prefusion S proteins to enhance the binding of SARS‐CoV‐2 virus on cell surface ACE2 and with the aid of cellular TMPRSS2, enhanced infection efficiency. Moreover, infection of SARS‐CoV‐2 preincubated with VCaP‐sEV that also contains activated TMPRSS2 and ACE2, in TMPRSS2‐expressing Calu‐3 cells and TMPRSS2‐non‐expressing VERO‐E6 and A549 cells, showed opposite outcomes. The significant decrease of SARS‐CoV‐2 infection in VERO‐E6 and A549 cells unravelled the importance of cellular TMPRSS2, in which the study showed SARS‐CoV‐2 entered target cells using the ‘early’ TMPRSS2‐dependent pathway instead of the ‘late’ cathepsin‐dependent endosomal membrane fusion route. Similarly, reduced SARS‐CoV‐2 infection pre‐treated with 293FT‐sEVs containing ACE2 and activated TMPRSS2 was also observed in VERO‐E6 cells in another study (Cocozza et al., 2020).

TMPRSS2 expression was shown to dictate the entry route used by SARS‐CoV‐2 to infect host cells (Icho et al., 2022; Koch et al., 2021). In the presence of cellular TMPRSS2, the proteolytic process of SARS‐CoV‐2 was completed at the plasma membrane and the virus rapidly entered the cells within 10 min in a pH‐dependent manner (Koch et al., 2021). In target cells that lacked TMPRSS2 expression, the virus was endocytosed and entered the cells via acid‐activated cathepsin L proteases 40–60 min post‐infection (Koch et al., 2021). In summary, SARS‐CoV‐2 infects cells through distinct, mutually exclusive entry routes and TMPRSS2 decides SARS‐CoV‐2 sorting into either pathway. In fact, TMPRSS2‐non‐expressing cells did not respond to Camostat (TMPRSS2 inhibitor) treatment to inhibit SARS‐CoV‐2 infection (Icho et al., 2022; Koch et al., 2021). Conversely, V‐ATPase inhibitor Bafilomycin A1 actively inhibits SARS‐CoV‐2 infection irrespective of TMPRSS2 expression (Icho et al., 2022; Koch et al., 2021).

Intriguingly, our previous study showed that 293T cell‐derived sEVs with no activated TMPRSS2 (only full‐length TMPRSS2 was detected) enhance the SARS‐CoV‐2 infection in VERO‐E6 cells (Tey et al., 2022), opposing to Berry et al. and Cocozza et al. studies (Berry et al., 2022; Cocozza et al., 2020). We postulate that the disparity could be due to the absence of activated TMPRSS2 in sEVs and the use of ‘late’ endosomal route for viral entry. In our data, ACE2‐enriched sEVs with no activated TMPRSS2 bind with SARS‐CoV‐2 virus and enter the cells through endocytosis. This hypothesis is supported by the fact that Bafilomycin A1 treatment reduces the infection efficacy of SARS‐CoV‐2 virus preincubated with ACE2‐sEVs in our study (Tey et al., 2022). It would be very interesting to investigate the effect of Bafilomycin A1 and Camostat on SARS‐CoV‐2 infection preincubated with HNECs‐derived mu‐sEV in both TMPRSS2‐expressing and ‐non‐expressing cells to understand the importance of sEV‐activated TMPRSS2 in dictating the entry route of SARS‐CoV‐2. Also, it would be relevant to test if sEV with no activated TMPRSS2 could enhance the SARS‐CoV‐2 infection in TMPRSS2‐expressing cells, as we only performed the infection in TMPRSS2‐non‐expressing VERO‐E6 cells. Importantly, a recent study has reported a decreased reliance on TMPRSS2 and a concurrent increased dependence on the endosomal route of entry by the Omicron variant, compared to other variants (Meng et al., 2022). In the study, Berry et al. only tested the proviral effect of VCaP‐sEV in SARS‐CoV‐2 wild‐type and Alpha variant. Therefore, testing the effect of such sEV in different SARS‐CoV‐2 variants could decipher the mechanistic pathways in great details.

Certainly, difference in the activation status of TMPRSS2 in sEVs between ours, Berry et al. and Cocozza et al. studies could be ascribed to the source of origin (293T, VCaP and 293FT cells). Different experimental designs are also found in these three studies. For virus infection assay, Cocozza et al. used pseudoviruses expressed by lentiviral‐based vector, which is less physiologically relevant compared to authentic viruses used in our and Berry et al. studies. Importantly, the incubation condition for sEVs and SARS‐CoV‐2 virus, and the viral infection time vary across studies. Berry et al. incubated viral particles (∼1.5 PFU/ml) with sEV (1 × 10^7^ − 1 × 10^9^ particles/ml) overnight at 4°C under gentle agitation, infected for 2–4 h at 37°C and assessed intracellular SARS‐CoV‐2 RNA 24–72 h post‐infection. Our study incubated viral particles with sEV for 1 h at room temperature, infected for 1 h at 37°C and assessed intracellular SARS‐CoV‐2 RNA at 1‐ and 24‐h post‐infection. The divergence between studies could also arise from the choice of cell lines to perform viral infection. Our study deployed the widely used VERO‐E6 cells, whereas Berry et al. and Cocozza et al. used Caco‐2, Calu‐3 and A549 cells. It is noteworthy that these cell lines have been shown previously to have a very low susceptibility to SARS‐CoV‐2 infection compared to VERO‐E6 cells (Yeung et al., 2021).

Another interesting postulation made by Berry et al. in the current report is the proviral effect seems to be specific for upper airway epithelial mu‐sEV as another study showed that sEV of salivary origin has no effect on SARS‐CoV‐2 infection (Conzelmann et al., 2020). However, as the status of ACE2 and activated TMPRSS2 is not confirmed in EVs of salivary origin, the observations could not be explained in more details. We hope that in the future, more evidence surface to explain the relationship between sEV and SARS‐CoV‐2 and their role in viral tropism.

Thus, combining the study by Berry et al. and ours, the status of activated TMPRSS2 within sEV could serve as a potential selective marker whether these sEVs are fit as therapeutic tools against SARS‐CoV‐2 infection. One should, therefore, still remain cautious when suggesting sEV as a therapeutic option for COVID‐19 as more comprehensive studies are needed to fully understand the multi‐faceted role of EVs in SARS‐CoV‐2 infection.

## References

[jev212296-bib-0001] Berry, F. , Morin‐Dewaele, M. , Majidipur, A. , Jamet, T. , Bartier, S. , Ignjatovic, E. , Toniutti, D. , Gaspar Lopes, J. , Soyeux‐Porte, P. , Maillé, P. , Saldana, C. , Brillet, R. , Ahnou, N. , Softic, L. , Couturaud, B. , Huet, É. , Ahmed‐Belkacem, A. , Fourati, S. , Louis, B. , … Bruscella, P. (2022). Proviral role of human respiratory epithelial cell‐derived small extracellular vesicles in SARS‐CoV‐2 infection. Journal of Extracellular Vesicles, 11, e12269. 36271885 10.1002/jev2.12269 PMC9587708

[jev212296-bib-0002] Ching, K. L. , De Vries, M. , Gago, J. , Dancel‐Manning, K. , Sall, J. , Rice, W. J. , Barnett, C. , Khodadadi‐Jamayran, A. , Tsirigos, A. , Liang, F.‐X. , Thorpe, L. E. , Shopsin, B. , Segal, L. N. , Dittmann, M. , Torres, V. J. , & Cadwell, K. (2022). ACE2‐containing defensosomes serve as decoys to inhibit SARS‐CoV‐2 infection. PLoS Biology, 20, e3001754. 36099266 10.1371/journal.pbio.3001754 PMC9469972

[jev212296-bib-0003] Cocozza, F. , Névo, N. , Piovesana, E. , Lahaye, X. , Buchrieser, J. , Schwartz, O. , Manel, N. , Tkach, M. , Théry, C. , & Martin‐Jaular, L. (2020). Extracellular vesicles containing ACE2 efficiently prevent infection by SARS‐CoV‐2 spike protein‐containing virus. Journal of Extracellular Vesicles, 10, e12050. 33391636 10.1002/jev2.12050 PMC7769856

[jev212296-bib-0004] Conzelmann, C. , Groß, R. , Zou, M. , Krüger, F. , Görgens, A. , Gustafsson, M. O. , El Andaloussi, S. , Münch, J. , & Müller, J. A. (2020). Salivary extracellular vesicles inhibit Zika virus but not SARS‐CoV‐2 infection. Journal of Extracellular Vesicles, 9, 1808281. 32939236 10.1080/20013078.2020.1808281 PMC7480612

[jev212296-bib-0005] El‐Shennawy, L. , Hoffmann, A. D. , Dashzeveg, N. K. , Mcandrews, K. M. , Mehl, P. J. , Cornish, D. , Yu, Z. , Tokars, V. L. , Nicolaescu, V. , Tomatsidou, A. , Mao, C. , Felicelli, C. J. , Tsai, C.‐F. , Ostiguin, C. , Jia, Y. , Li, L. , Furlong, K. , Wysocki, J. , Luo, X. , … Liu, H. (2022). Circulating ACE2‐expressing extracellular vesicles block broad strains of SARS‐CoV‐2. Nature Communications, 13, 405. 10.1038/s41467-021-27893-2 PMC8776790 35058437

[jev212296-bib-0006] Icho, S. , Rujas, E. , Muthuraman, K. , Tam, J. , Liang, H. , Landreth, S. , Liao, M. , Falzarano, D. , Julien, J.‐P. , & Melnyk, R. A. (2022). Dual inhibition of vacuolar‐ATPase and TMPRSS2 is required for complete blockade of SARS‐CoV‐2 entry into cells. Antimicrobial Agents and Chemotherapy, 66, e0043922. 35703551 10.1128/aac.00439-22 PMC9295568

[jev212296-bib-0007] Inal, J. M. (2020). Decoy ACE2‐expressing extracellular vesicles that competitively bind SARS‐CoV‐2 as a possible COVID‐19 therapy. Clinical Science (London, England: 1979), 134, 1301–1304. 32542396 10.1042/CS20200623 PMC7298154

[jev212296-bib-0008] Kim, H. K. , Cho, J. , Kim, E. , Kim, J. , Yang, J.‐S. , Kim, K.‐C. , Lee, J.‐Y. , Shin, Y. , Palomera, L. F. , Park, J. , Baek, S. H. , Bae, H.‐G. , Cho, Y. , Han, J. , Sul, J. H. , Lee, J. , Park, J. H. , Cho, Y. W. , Lee, W. , & Jo, D.‐G. (2022). Engineered small extracellular vesicles displaying ACE2 variants on the surface protect against SARS‐CoV‐2 infection. Journal of Extracellular Vesicles, 11, e12179. 34982509 10.1002/jev2.12179 PMC8725171

[jev212296-bib-0009] Koch, J. , Uckeley, Z. M. , Doldan, P. , Stanifer, M. , Boulant, S. , & Lozach, P.‐Y. (2021). TMPRSS2 expression dictates the entry route used by SARS‐CoV‐2 to infect host cells. EMBO Journal, 40, e107821. 34159616 10.15252/embj.2021107821 PMC8365257

[jev212296-bib-0010] Meng, B. , Abdullahi, A. , Ferreira, I. A. T. M. , Goonawardane, N. , Saito, A. , Kimura, I. , Yamasoba, D. , Gerber, P. P. , Fatihi, S. , Rathore, S. , Zepeda, S. K. , Papa, G. , Kemp, S. A. , Ikeda, T. , Toyoda, M. , Tan, T. S. , Kuramochi, J. , Mitsunaga, S. , Ueno, T. , … Gupta, R. K. (2022). Altered TMPRSS2 usage by SARS‐CoV‐2 Omicron impacts infectivity and fusogenicity. Nature, 603, 706–714. 35104837 10.1038/s41586-022-04474-x PMC8942856

[jev212296-bib-0011] Rao, L. , Xia, S. , Xu, W. , Tian, R. , Yu, G. , Gu, C. , Pan, P. , Meng, Q.‐F. , Cai, X. , Qu, D. , Lu, L. , Xie, Y. , Jiang, S. , & Chen, X. (2020). Decoy nanoparticles protect against COVID‐19 by concurrently adsorbing viruses and inflammatory cytokines. Proceedings of the National Academy of Sciences of the United States of America, 117, 27141–27147. 33024017 10.1073/pnas.2014352117 PMC7959535

[jev212296-bib-0012] Tey, S. K. , Lam, H. , Wong, S. W. K. , Zhao, H. , To, K. K.‐W. , & Yam, J. W. P. (2022). ACE2‐enriched extracellular vesicles enhance infectivity of live SARS‐CoV‐2 virus. Journal of Extracellular Vesicles, 11, e12231. 35582880 10.1002/jev2.12231 PMC9115585

[jev212296-bib-0013] Wu, C. , Xu, Q. , Wang, H. , Tu, B. , Zeng, J. , Zhao, P. , Shi, M. , Qiu, H. , & Huang, Y. (2022). Neutralization of SARS‐CoV‐2 pseudovirus using ACE2‐engineered extracellular vesicles. Acta Pharmaceutica Sinica B, 12, 1523–1533. 34522576 10.1016/j.apsb.2021.09.004 PMC8427979

[jev212296-bib-0014] Xie, F. , Su, P. , Pan, T. , Zhou, X. , Li, H. , Huang, H. , Wang, A. , Wang, F. , Huang, J. , Yan, H. , Zeng, L. , Zhang, L. , & Zhou, F. (2021). Engineering extracellular vesicles enriched with palmitoylated ACE2 as COVID‐19 therapy. Advanced Materials, 33, 2103471. 34665481 10.1002/adma.202103471 PMC8646473

[jev212296-bib-0015] Yeung, M. L. , Teng, J. L. L. , Jia, L. , Zhang, C. , Huang, C. , Cai, J.‐P. , Zhou, R. , Chan, K.‐H. , Zhao, H. , Zhu, L. , Siu, K.‐L. , Fung, S.‐Y. , Yung, S. , Chan, T. M. , To, K. K.‐W. , Chan, J. F.‐W. , Cai, Z. , Lau, S. K. P. , Chen, Z. , … Yuen, K.‐Y. (2021). Soluble ACE2‐mediated cell entry of SARS‐CoV‐2 via interaction with proteins related to the renin‐angiotensin system. Cell, 184, 2212.e12–2228.e12. 33713620 10.1016/j.cell.2021.02.053 PMC7923941

[jev212296-bib-0016] Zhang, J. , Huang, F. , Xia, B. , Yuan, Y. , Yu, F. , Wang, G. , Chen, Q. , Wang, Q. , Li, Y. , Li, R. , Song, Z. , Pan, T. , Chen, J. , Lu, G. , & Zhang, H. (2021). The interferon‐stimulated exosomal hACE2 potently inhibits SARS‐CoV‐2 replication through competitively blocking the virus entry. Signal Transduction and Targeted Therapy, 6, 189. 33980808 10.1038/s41392-021-00604-5 PMC8113286

[jev212296-bib-0017] Zhang, Q. , Jeppesen, D. K. , Higginbotham, J. N. , Franklin, J. L. , Crowe, J. E. Jr. , & Coffey, R. J. (2021). Angiotensin‐converting enzyme 2‐containing small extracellular vesicles and exomeres bind the Severe Acute Respiratory Syndrome Coronavirus 2 spike protein. Gastroenterology, 160, 958.e3–961.e3. 33022277 10.1053/j.gastro.2020.09.042 PMC7832655

